# Revision of the *Conwentzia* Enderlein, 1905 (Neuroptera, Coniopterygidae) of China, combining morphological and molecular characters

**DOI:** 10.3897/zookeys.1026.61700

**Published:** 2021-03-25

**Authors:** Yaru Zhao, Ying Li, Zhiqi Liu

**Affiliations:** 1 Department of Entomology, China Agricultural University, Beijing 100094, China China Agricultural University Beijing China

**Keywords:** DNA barcoding, dusty lacewings, faunistics, identification key, taxonomy

## Abstract

The Chinese species of *Conwentzia* Enderlein are revised by integrating morphological characters and molecular data. *Conwentzia yunguiana* Liu & Yang, 1993 is proposed as a junior synonym of *Conwentzia nietoi* Monserrat, 1982, **syn. nov.** and *Conwentzia orthotibia* Yang, 1974 is proposed as a junior synonym of *Conwentzia pineticola* Enderlein, 1905, **syn. nov.** Moreover, a key to the adult males of the *Conwentzia* from China and DNA barcodes are provided.

## Introduction

The dusty lacewing genus *Conwentzia* belongs to the subfamily Coniopteryginae of family Coniopterygidae and it is a species-poor genus, including only fourteen described species (Sziráki 2011; Oswald 2020). However, *Conwentzia* is relatively widespread, and widely distributed in the Palaearctic, Nearctic, Oriental and Afrotropical regions (Meinander 1972; Sziráki 2011). This genus was originally erected by Enderlein (1905) based on the type species *Conwentzia pineticola* Enderlein, 1905. All *Conwentzia* species are easily set apart from other Coniopteryginae due to the highly reduced hind wings, except the North American species *C. barretti* (Banks 1899), which has fully developed hind wings (Meinander 1972; Sziráki 2011). Like other dusty lacewings, *Conwentzia* species are predators of small phytophagous arthropods – including notorious pests such as phylloxerids and tetranychid mites – and are thus potential biocontrol agents, being quite common in orchards, especially on *Citrus* (Collyer 1951; Meinander 1972; Pantaleoni 2007). Consequently, it is necessary to effectively identify *Conwentzia* species. Although *Conwentzia* species can be identified by male genitalia, it is still hard to identify their females and larvae. At the same time, type material is not easily accessible for all students of this genus. These problems highlight the necessity for accurate and easy methods to identify *Conwentzia* species.

DNA barcoding is a useful tool to identify species (Hebert et al. 2003). In many insects, the partial mitochondrial cytochrome c oxidase subunit I (COI) gene is an effective marker (Raupach et al. 2020), and it can also be used for dusty lacewings (Morinière et al. 2014; Yi et al. 2018). We collected some fresh specimens of *Conwentzia* species from China in the past three years, and decided to provide DNA barcodes for these species.

In China, there are four species of *Conwentzia* (Sziráki 2011; Oswald 2020). However, *Conwentzia fraternalis* Yang, 1974 is only known based on a single female and its status remains enigmatic. The aim of the present paper is to review the three remaining Chinese species of *Conwentzia* using morphological characters and molecular data.

## Material and methods

### Morphological comparisons

The examined specimens are preserved in 95% ethyl alcohol and deposited in the Entomological Museum of China Agricultural University, Beijing (**CAU**). The abdomen was dissected from the body and macerated in a heated solution of 5% KOH for 5 minutes, then rinsed in water and 95% ethyl ethanol. The cleared abdomen was transferred to glycerol for dissection and study. After examination, the abdomen was placed in glycerol and in a 200 μL microtube for long-term preservation, while the head and thorax were placed in 95% ethyl alcohol and in another 200 μL microtube. The two 200 μL microtubes were then placed in a 5 mL microtube at -20°C.

### Terminology

Morphological terminology mostly follows Meinander (1972) for general morphology and Aspöck and Aspöck (2008) for male genitalia.

### Imaging

Specimens were examined with an Optec SZ760 stereomicroscope. Photos were taken with a Nikon D5300 digital camera attached to a Leica DM2500 stereomicroscope. The resulting images were edited and processed with Adobe Photoshop CC 2018.

### DNA extraction and sequencing protocols

Total genomic DNA was extracted based on the method of Lu et al. (2018) with the commercial Ezup Column Animal Genomic DNA Purification Kit (Sangon Biotech, China) and following the manufacturer’s protocol. The PCR primer and reaction conditions for the COI region followed Folmer et al. (1994) and Lu et al. (2018). Products were sequenced in a single direction by Sangon (Shanghai) Co., Ltd. Sequences were edited and analyzed using the software Chromas version 2.3 and BioEdit 7.0.4.1 (Hall 1999). In addition, sequences were translated into amino acids to check for NUMTS and test for quality.

### Sequence analysis

The barcoding gap was assessed by means of the following methods. Pairwise genetic distances for COI genes were computed with the Kimura 2-parameter (K2P) method in the MEGA 6.0 software (Tamura et al. 2011). Finally, all sequences were deposited in GenBank. In order to better analyze the data, sequences of *C. pineticola* from Bavaria in Germany were downloaded from GenBank. The accession numbers of these sequences are as follows: JN299372, JN299373, JN299374, JN299347, JN299348.

## Results

### Morphological characters

#### Genus Conwentzia

Taxon classificationAnimaliaNeuropteraConiopterygidae

Enderlein, 1905

6F8208BE-B048-58E6-9E20-A2DF31F230A7

##### Type species.

*Conwentzia pineticola* Enderlein, 1905

##### Diagnosis.

Fore wing with RP vein forked. Hind wing reduced except in *C. barretti*. Male genitalia with gonocoxites 9 absent, gonapophyses 9 (when present) originating from sclerotized ring of segment 9.

##### Comments.

There are fourteen species in the genus *Conwentzia*. The species described before 1972 are well known thanks to Meinander’s (1972) comprehensive revision. However, *Conwentzia inverta* Withycombe, 1925 was not redescribed by Meinander (1972) because the type material in the Natural History Museum, London is in rather bad condition (Meinander 1972). However, Monserrat found that Barnard had a specimen collected from the type locality, Pusa, in India, by Withycombe in 1925, which he examined and used for his redescription of *C. inverta* (Monserrat 1982). Species described after 1972 are well known thanks to Sziráki’s (2011) comprehensive revision. Thus, all species in the genus *Conwentzia* are relatively well known.

### Key to Chinese species of *Conwentzia* (males)

Note: *Conwentzia fraternalis* Yang, 1974 is not included in the key as the specimen is only known based on a single female.

**Table:** 

1	Gonocoxites 9 (inner process of ectoprocts *sensu*Meinander 1972) present (Fig. 6c–f)	***C. pineticola* Enderlein**
–	Gonocoxites 9 absent (Figs 2c–f, 4c–f)	**2**
2	Gonapophyses 9 (stylus *sensu*Meinander 1972) short basally (Fig. 2a, b)	***C. sinica* Yang**
–	Gonapophyses 9 long and slender (Fig. 4a, b)	***C. nietoi* Monserrat**

#### Conwentzia sinica

Taxon classificationAnimaliaNeuropteraConiopterygidae

Yang, 1974

57F41645-D812-555F-913E-1DD8E7178DEE

Figs 1 , 2

Conwentzia sinica Yang, 1974: 84. Type locality: China (Shaanxi).

##### Type material examined.

***Holotype*:** male (CAU), China: Shaanxi (Province): Xian (City): Zhouzhi (County), [34.0588°N, 108.3371°E], 13–18.viii.1962, leg. Chikun Yang and Fasheng Li.

##### Other material examined.

14 males and 31 females (CAU), China: Zhejiang (Province): Jiaxing (City): Wuzhen (Town), [30.7509°N, 120.5024°E], 18.v.2018, leg. Zhiqi Liu. 13 males and 26 females (CAU), China: Yunnan (Province): Kunming (City), [25.1371°N, 102.7493°E], 31.vii.2019, leg. Yaru Zhao and Ying Li. 5 males and 9 females (CAU), China: Jilin (Province): Yanji (City), [42.9057°N, 129.4955°E], 1.viii.2019, leg. Yaru Zhao and Ying Li. 5 males and 7 females (CAU), China: Jilin (Province): Yanji (City), [43.8293°N, 126.5253°E], 2.viii.2019, leg. Yaru Zhao and Ying Li. 5 males and 9 females (CAU), China: Jilin (Province): Yanji (City), [42.9057°N, 129.4955°E], 1.viii.2019, leg. Yaru Zhao and Ying Li. 4 males and 6 females (CAU), China: Shanghai (City), [31.2118°N, 121.4981°E], 14.xi.2019, leg. Mingming Zou. 46 males and 83 females (CAU), China: Shaanxi (Province): Xian (City): Zhouzhi (County), [34.0588°N, 108.3371°E], 13–18.viii.1962, leg. Chikun Yang and Fasheng Li. 1 male and 1 female (CAU), China: Shaanxi (Province): Xi’an (City): Qinling (Mountain), [33.9717°N, 109.0112°E], 5–7.viii.1962, leg. Chikun Yang and Fasheng Li.

##### Diagnosis.

Gonocoxites 9 absent; gonocoxites 11 (tenth sternite) forming a parallelogram in lateral view; gonapophyses 9 slender in caudal view.

##### Redescription.

***Measurements*.** Forewing length 2.5–3.4 mm, width 0.9–1.5 mm. Hindwing reduced; length 1.0–1.6 mm, width 0.4–0.6 mm.

**Figure 1.**
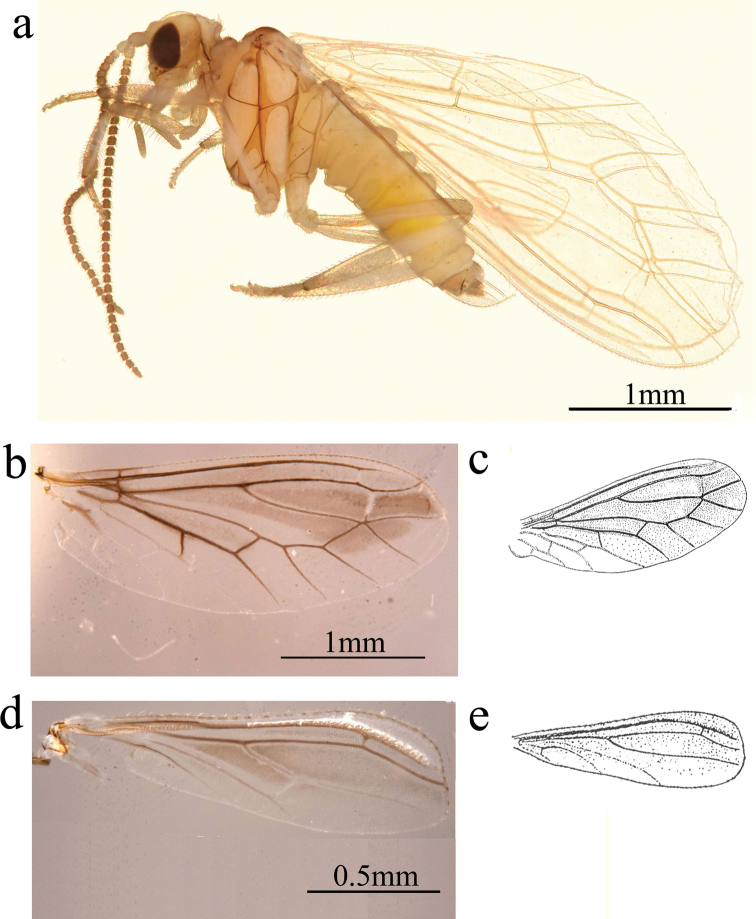
*Conwentzia sinica* Yang, 1974, male **a** habitus, lateral view **b, c** fore wing **d, e** hind wing.

***Head*** (Fig. 1a). Yellowish brown. Compound eyes large and dark. Antennae brown, 31–36-segmented. Scape relatively broad and blunt. Pedicel cylindrical and longer than wide. Scape and pedicel light brown. Flagellomeres dark brown. Maxillary and labial palps brown.

***Thorax*.** Brown. Nota with dorsal dark spots. Legs brown.

***Wing*** (Fig. 1b–e). Wing membrane almost hyaline, light greyish brown.

**Figure 2.**
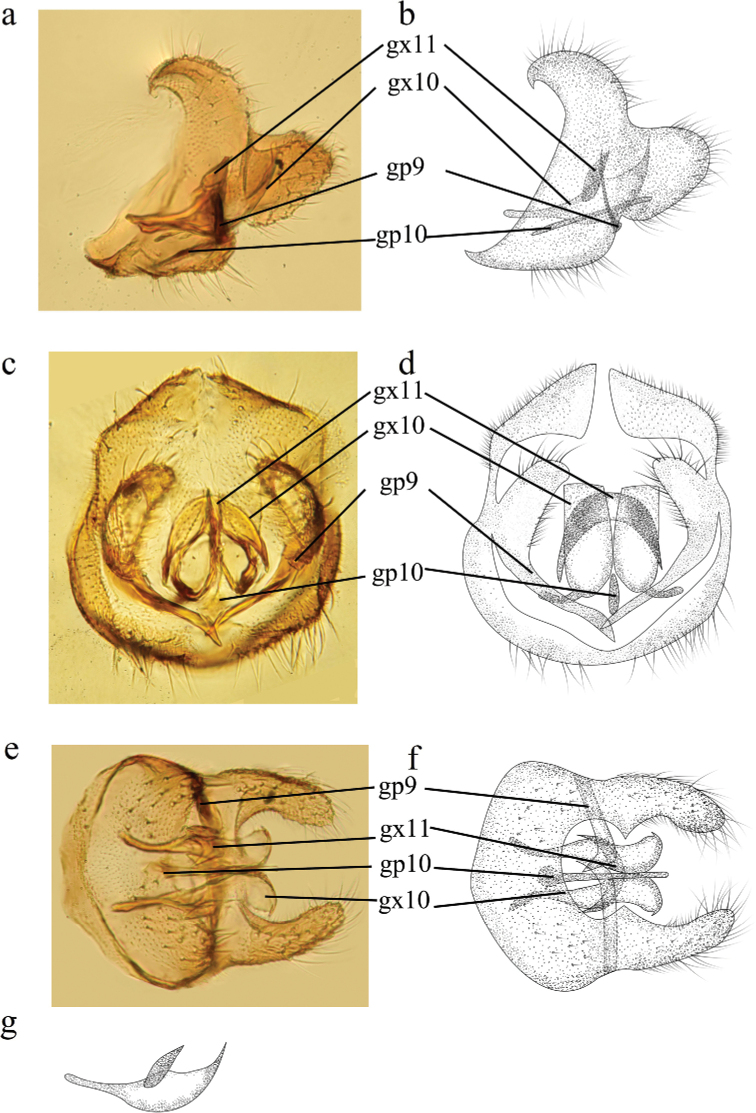
*Conwentzia sinica* Yang, 1974, male genitalia **a, b** lateral view **c, d** caudal view **e, f** ventral view **g** gonocoxites 10, lateral view.

***Male genitalia*** (Fig. 2a–g). Outer process of ectoprocts relatively broad in lateral view. Gonocoxites 9 (inner process of ectoprocts *sensu*Meinander 1972) absent. Gonapophyses 9 (stylus *sensu*Meinander 1972) short with a hook in distal part ventrally (Fig. 2e–f). Gonocoxites 10 (paramere *sensu*Meinander 1972) relatively short in basal part, median section wider and stout, distal section bent upward in lateral view (Fig. 2a, b). Gonapophyses 10 (penis *sensu*Meinander 1972) slender and swollen at base in ventral view (Fig. 2e, f). Gonocoxites 11 (tenth sternite *sensu*Meinander 1972) sub-rectangular in lateral view (Fig. 2a, b).

##### Distribution.

China (Hebei, Shanxi, Liaoning, Jilin, Jiangsu, Zhejiang, Fujian, Guangdong, Guangxi, Yunnan, Shaanxi, Gansu).

#### Conwentzia nietoi

Taxon classificationAnimaliaNeuropteraConiopterygidae

Monserrat, 1982

220F7B18-E179-512C-8EA0-EB30858FF1B7

Figs 3 , 4

Conwentzia nietoi Monserrat, 1982: 24. Type locality: Sri Lanka (Colombo).
Conwentzia yunguiana Liu & Yang, 1993: 256. Type locality: China (Guizhou: Guiyang). Syn. nov.

##### Type material examined.

***Holotype*** of *Conwentzia yunguiana*: male (CAU), China: Guizhou (Province): Guiyang (City), [26.5003°N, 106.7467°E], 29.viii.1987, leg. Hongye Chen. ***Paratypes***: 3 males, same data as holotype (CAU).

##### Other material examined.

1 male (CAU), China: Guizhou (Province): Libo (County), [25.2681°N, 108.0780°E], 18.v.1990, leg. Chunqing Yang. 1 male (CAU), China: Yunan (Province): Jinghong (City), [21.8566°N, 100.9582°E], 12.ix.1989, leg. Fasheng Li. 1 male (CAU), China: Guangxi (Province): Lingui (City): Yanshan (District), [24.9265°N, 110.5040°E], 13.vi.1982, leg. Fasheng Li. 1 male (CAU), China: Sichuan (Province): Leshan (City): E’meishan (Mountain), [29.5738°N, 103.3563°E], 15.iv.1990, leg. Chunqing Yang and Zhiqi Liu.

##### Diagnosis.

Gonocoxites 9 absent; gonocoxites 11 subtriangular in lateral view; gonapophyses 9 basally broad and blunt in caudal view.

##### Redescription.

***Measurements*.** Forewing length 2.5–3.2 mm, width 0.9–1.3 mm. Hindwing reduced; length 1.0–1.5 mm, width 0.2–0.3 mm.

**Figure 3.**
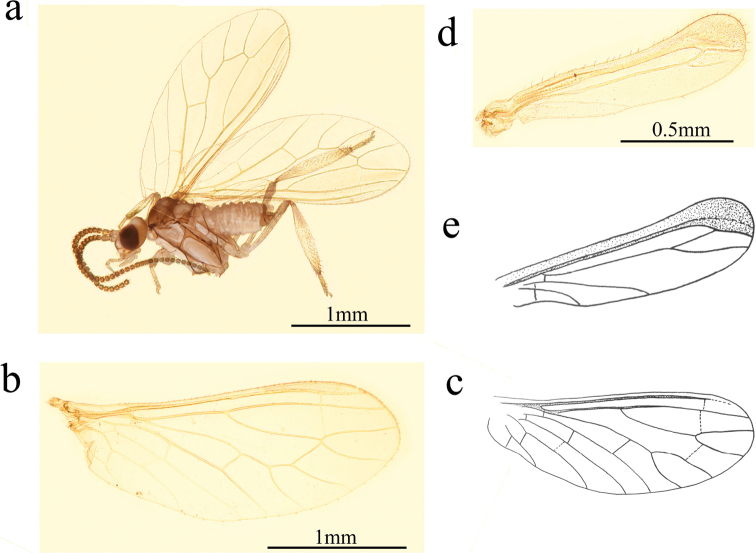
*Conwentzia nietoi* Monserrat, 1982, male **a** habitus, lateral view **b, c** fore wing **d, e** hind wing.

***Head*** (Fig. 3a). Brown. Compound eyes large and dark. Antennae brown (except light brown scape), 34–35-segmented. Scape broad and blunt. Pedicel cylindrical and longer than broad. Antennae entirely brown, scape light brown. Maxillary and labial palps brown.

***Thorax*.** Yellowish-brown. Nota with dorsal dark spots. Legs brown.

***Wing*** (Fig. 3b–e). Wing membrane almost hyaline, light greyish brown.

**Figure 4.**
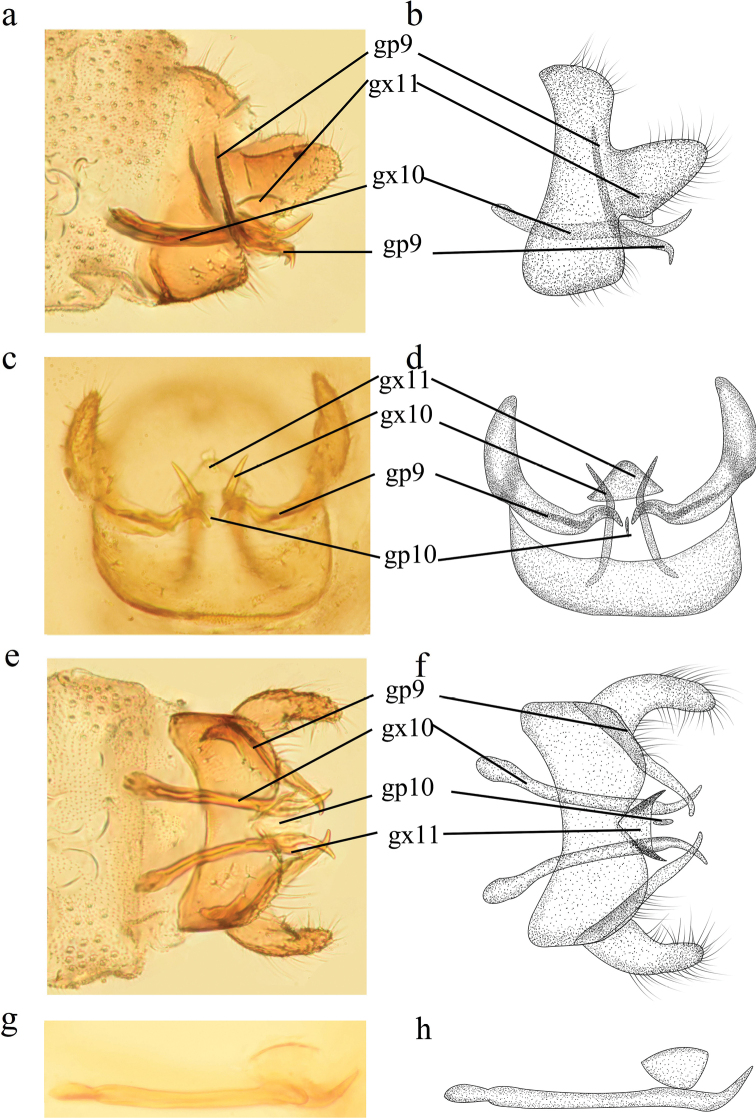
*Conwentzia nietoi* Monserrat, 1982, male genitalia **a, b** lateral view **c, d** caudal view **e, f** ventral view **g, h** gonocoxites 10, lateral view.

***Male genitalia*** (Fig. 4a–h). Outer process of ectoprocts finger-like in ventral view, longer than wide in lateral view (Fig. 4a, b). Gonocoxites 9 absent. Gonapophyses 9 hooked downward in lateral view (Fig. 4a, b). Gonocoxites 10 slender, distal section bent upward in lateral view (Fig. 4a, b). Gonapophyses 10 small and short in ventral view (Fig. 4e, f). Gonocoxites 11 subtriangular in caudal view (Fig. 4c, d) and ventral view (Fig. 4e, f).

##### Distribution.

China (Guangxi, Sichuan, Guizhou, Yunnan).

#### Conwentzia pineticola

Taxon classificationAnimaliaNeuropteraConiopterygidae

Enderlein, 1905

01F2007A-F42C-51D9-AB1F-DA3ACDB268AB

Figs 5 , 6

Conwentzia pineticola Enderlein, 1905: 10. Type locality: Germany (Berlin).
Conwentzia orthotibia Yang, 1974: 88. Type locality: China (Gansu: Longnan). Syn. nov.

##### Type material examined.

***Holotype*** of *Conwentzia orthotibia*: male (CAU), China: Gansu (Province): Longnan (city): Wudu (District), [33.3740°N, 104.9665°E], 25.vi.1958, leg. Xuemin Zhang. ***Paratype***: 1 male, same data as holotype (CAU).

##### Other material examined.

7 males and 10 females (CAU), China: Gansu (Province): diebu (County), [34.1286°N, 106.5364°E], 9.vii.2017, leg. Yaru Zhao and Mingwei Ma. 23 males and 30 females (CAU), China: Gansu (Province): Zhangye (City), [34.1669°N, 106.5400°E], 13.vii.2017, leg. Yaru Zhao and Mingwei Ma. 5 males and 6 females (CAU), China: Gansu (Province): diebu (County), [33.9583°N, 103.5506°E], 13. vii.2017, leg. Yaru Zhao and Mingwei Ma. 1 male (CAU), China: Liaoning (Province): Dandong (City), [40.1247°N, 124.3928°E], 27.vii.2017, leg. Yaru Zhao and Ying Li. 1 male and 3 females (CAU), China: Liaoning (Province): Dandong (City), [40.1247°N, 124.3928°E], 28.vii.2017, leg. Yaru Zhao and Ying Li. 5 males and 4 females (CAU), China: Sichuan (Province): Panzhihua (City), [25.0120°N, 98.4800°E], 3.iv.2019, leg. Yaru Zhao and Mingming Zou.

##### Diagnosis.

Gonocoxites 9 present; gonocoxites 11 rod-shaped in lateral view; gonapophyses 9 basally broad in caudal view.

##### Redescription.

***Measurements*.** Forewing length 3.1–3.5 mm, width 1.1–1.4 mm. Hindwing reduced; length 1.3–1.4 mm, width 0.5–0.6 mm.

**Figure 5.**
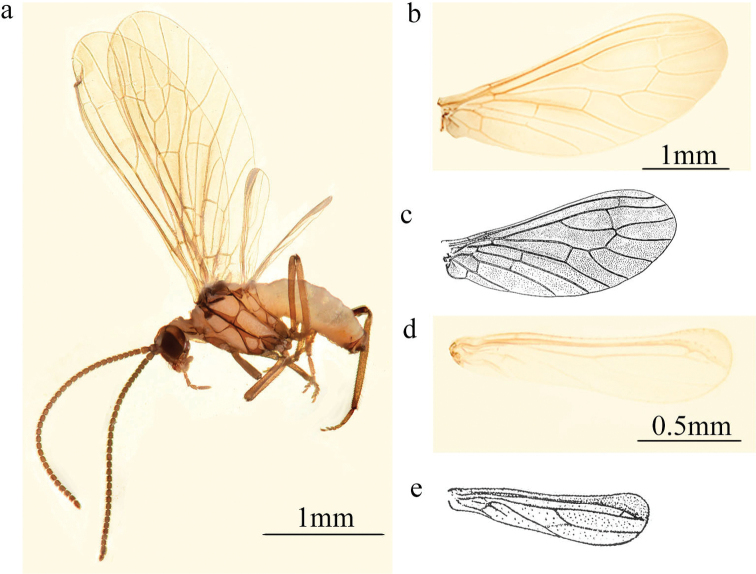
*Conwentzia pineticola* Enderlein, 1905, male **a** habitus, lateral view **b, c** fore wing **d, e** hind wing.

***Head*** (Fig. 5a). Yellowish-brown. Compound eyes large and dark. Antennae 36–37-segmented in males and 32–36-segmented in females. Scape relatively broad and blunt. Pedicel cylindrical, longer than wide. Antennae brown; scape light brown in some specimens. Scape broad and blunt. Pedicel cylindrical and longer than broad. Maxillary and labial palpus brown.

***Thorax*.** Brown. Nota with dorsal dark spots. Legs brown.

***Wing*** (Fig. 5b–e). Wing membrane almost hyaline, light greyish brown.

**Figure 6.**
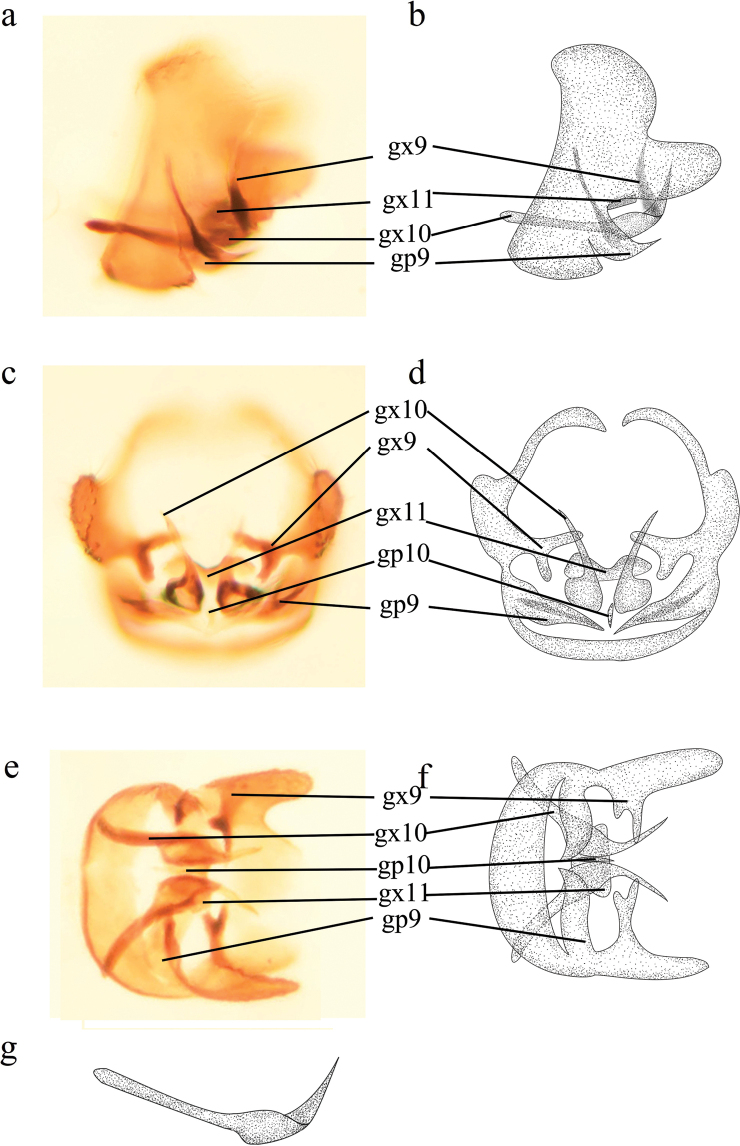
*Conwentzia pineticola* Enderlein, 1905, male genitalia **a, b** lateral view **c, d** caudal view **e, f** ventral view **g** gonocoxites 10, lateral view.

***Male genitalia*** (Fig. 6a–g). Outer process of ectoprocts finger-like in lateral view (Fig. 6a, b). Gonocoxites 9 furcate, with dorsal branch slightly longer than ventral one in caudal view (Fig. 6c, d). Gonapophyses 9 broad, distally hooked in ventral view (Fig. 6e, f). Gonocoxites 10 slender, distally bent upward in lateral view (Fig. 6a, b). Gonapophyses 10 short and straight in ventral view (Fig. 6e, f). Gonocoxites 11 subtriangular in ventral view (Fig. 6e, f).

##### Distribution.

China (Hebei, Shanxi, Jilin, Heilongjiang, Henan, Hubei, Chongqing, Sichuan, Yunnan, Tibet, Gansu, Qinghai, Ningxia, Xinjiang, Liaoning, Sichuan).

### Molecular data

#### DNA barcoding

For the three *Conwentzia* species treated in this paper, accession numbers of DNA barcodes are listed in Table 1.

**Table 1.** *Conwentzia* specimens used in this study, with information on locality, geographic coordinates, sex, GenBank accession numbers and voucher specimen numbers.

Species	Locality	Geographic coordinates	Sex	Accession number	Voucher specimen number
*C. sinica*	Ruili, Dehong, China	24.0723°N, 97.8174°E	Male	MW093443	CAU-CONIO-00000348
	Ruili, Dehong, China	24.0723°N, 97.8174°E	Male	MW093444	CAU-CONIO-00000349
	Ruili, Dehong,China	24.0723°N, 97.8174°E	Male	MW093445	CAU-CONIO-00000350
*C. nietoi*	Longchuan, Dehong, China	24.1776°N, 97.7947°E	Male	MW093440	CAU-CONIO-00000320
	Longchuan, Dehong, China	24.1776°N, 97.7947°E	Male	MW093441	CAU-CONIO-00000322
	Yuanjiang, Yuxi, China	23.6001°N, 102.0098°E	Male	MW093442	CAU-CONIO-00000374
*C. pineticola*	Diebu, Gannan, China	33.9583°N, 103.5506°E	Male	MW093437	CAU-CONIO-00000026
	Diebu, Gannan, China	33.9583°N, 103.5506°E	Male	MW093438	CAU-CONIO-00000027
	Diebu, Gannan, China	34.1286°N, 106.5364°E	Male	MW093439	CAU-CONIO-00000043
	Ganzhou, Zhangye, China	34.1669°N, 106.5400°E	Male	MW093435	CAU-CONIO-00000025
	Ganzhou, Zhangye, China	34.1669°N, 106.5400°E	Male	MW093436	CAU-CONIO-00000023
	Panzhihua, Sichuan, China	25.0120°N, 98.4800°E	Male	MW093434	CAU-CONIO-00000338

#### Genetic divergence among species

The average intraspecific genetic distance based on the K2P model was 0.10% for *Conwentzia sinica* Yang, 1974, 0.10% for *Conwentzia orthotibia* Yang, 1974, 0.10% for *Conwentzia yunguiana* Liu & Yang, 1993, and 0.52% for *C. pineticola*. The average interspecific genetic distance based on the K2P model was 2.19% between *C. orthotibia* and *C. pineticola*. The other average interspecific genetic distances based on K2P model were 11.14–14.54%. The results (Table 2) showed that all intraspecific genetic distances were less than 2.0%, and all the interspecific genetic distance values exceeded 10% (except for the *C. orthotibia* and *C. pineticola* genetic distance).

**Table 2.** Intra- and interspecific Kimura 2-parameter average divergence values (%) of the COI gene analyzed by the MEGA 6.0 software. * = sequences of *C. pineticola* from Bavaria in Germany downloaded from GenBank.

Species	*C. pineticola*	*C. sinica*	C. pineticola*	*C. nietoi*
*C. pineticola*	0.10	–	–	–
*C. sinica*	14.14	0.10	–	–
C. pineticola*	**2.19**	14.54	0.52	–
*C. nietoi*	12.93	11.14	12.70	0.10

## Discussion

*Conwentzia sinica* is similar to *C. inverta* but differs in the shape of the male genitalia. *Conwentzia sinica* is characterized by a slender gonapophyses 10 in lateral view (Fig. 2a, b), while it is short (Monserrat 1982: 26, fig. 39) in *C. inverta*, therefore *C. sinica* is three times longer than *C. inverta* for gonapophyses 10. Moreover, the basal part of gonapophyses 10 is broad and blunt ventrally in *C. sinica* but acute in *C. inverta*. Furthermore, gonocoxites 10 are rectangular caudally in *C. sinica* (Fig. 2c, d) but oval in *C. inverta* (Monserrat 1982: 26, fig. 38). The morphological differences between *C. sinica* and *C. inverta* are mainly centered around the gonapophyses 10 and gonocoxites 10. However, both structures are almost transparent, requiring careful examination.

For *C. yunguiana*, we found that those specimens do not have clear differences after comparison of the type specimens of *C. yunguiana* with the description of *Conwentzia nietoi* Monserrat, 1982. Nevertheless, the distal part of gonocoxites 11 is blunt laterally in *C. yunguiana* (Fig. 4a, b), while it is acute in *C. nietoi* (Monserrat 1982: 26, fig. 34). The differences are mainly centered around the distal part of gonocoxites 11 in lateral view, which may be caused by the arched shape above the gonocoxites 10 in lateral view. Besides, the rim is so obscure for the gonocoxites 11 is membranous and transparent in distal part. And we also discussed with György Sziráki, who examined the type specimen of *C. nietoi*, and his opinion is the same as ours. Therefore, we ascribe the differences in gonocoxites 11 to intraspecific morphological variation.

We found no clear morphological differences between *C. orthotibia* and *C. pineticola* after comparison of the type specimens of *C. orthotibia* with the description of *C. pineticola*. Nevertheless, the distal part of gonocoxites 11 is wavy caudally in *C. orthotibia* (Fig. 6c, d), while it is arched in *C. pineticola* (Meinander 1972: 300, fig. 195F). We were not sure whether such differences should be ascribed to intraspecific morphological variation between *C. orthotibia* and *C. pineticola*. The type species was described from Berlin in Germany (Enderlein 1905) and we obtained DNA barcodes of *C. pineticola* from Bavaria in Germany from NCBI. The results show that the mean interspecific divergence between *C. orthotibia* and *C. pineticola* was 2.19%, which is inconsistent with Morinière et al.’s (2014) suggestion that the mean interspecific divergence is 10–20% in the Coniopterygidae, Hemerobiidae, and Myrmeleontidae. Our results suggest that the differences between *C. orthotibia* and *C. pineticola* are intraspecific.

## Conclusion

*Conwentzia yunguiana* Liu & Yang, 1993 is proposed as a junior synonym of *Conwentzia nietoi* Monserrat, 1982, syn. nov. and *Conwentzia orthotibia* Yang, 1974 is proposed as a junior synonym of *Conwentzia pineticola* Enderlein, 1905, syn. nov. In this study, we added three species barcodes to the *Conwentzia* DNA library and the mean intraspecific divergence was 11.14–14.54% for the species analysed.

## Supplementary Material

XML Treatment for Conwentzia

XML Treatment for Conwentzia sinica

XML Treatment for Conwentzia nietoi

XML Treatment for Conwentzia pineticola
